# Chickpea Biofortification for Cytokinin Dehydrogenase *via* Genome Editing to Enhance Abiotic-Biotic Stress Tolerance and Food Security

**DOI:** 10.3389/fgene.2022.900324

**Published:** 2022-05-20

**Authors:** Rohit Kumar Mahto, Ambika, Charul Singh, B S. Chandana, Rajesh Kumar Singh, Shruti Verma, Vijay Gahlaut, Murli Manohar, Neelam Yadav, Rajendra Kumar

**Affiliations:** ^1^ Indian Agricultural Research Institute (ICAR), New Delhi, India; ^2^ Department of Genetics and Plant Breeding, UAS, Bangalore, India; ^3^ University School of Biotechnology, Guru Gobind Singh Indraprastha University, New Delhi, India; ^4^ NCoE-SAM, Department of Pediatrics, KSCH, Lady Hardinge Medical College, New Delhi, India; ^5^ Institute of Himalayan Bioresource Technology (CSIR), Palampur, India; ^6^ Boyce Thompson Institute, Cornell University, Ithaca, NY, United States; ^7^ Centre of Food Technology, University of Allahabad, Prayagraj, India

**Keywords:** biofortification, chickpea, cytokinindehydrogenase (CKX), genome-editing, stress

## Abstract

Globally more than two billion people suffer from micronutrient malnutrition (also known as “hidden hunger”). Further, the pregnant women and children in developing nations are mainly affected by micronutrient deficiencies. One of the most important factors is food insecurity which can be mitigated by improving the nutritional values through biofortification using selective breeding and genetic enhancement techniques. Chickpea is the second most important legume with numerous economic and nutraceutical properties. Therefore, chickpea production needs to be increased from the current level. However, various kind of biotic and abiotic stresses hamper global chickpea production. The emerging popular targets for biofortification in agronomic crops include targeting cytokinin dehydrogenase (*CKX*). The *CKXs* play essential roles in both physiological and developmental processes and directly impact several agronomic parameters i.e., growth, development, and yield. Manipulation of *CKX* genes using genome editing tools in several crop plants reveal that *CKXs* are involved in regulation yield, shoot and root growth, and minerals nutrition. Therefore, *CKXs* have become popular targets for yield improvement, their overexpression and mutants can be directly correlated with the increased yield and tolerance to various stresses. Here, we provide detailed information on the different roles of *CKX* genes in chickpea. In the end, we discuss the utilization of genome editing tool clustered regularly interspaced short palindromic repeats/CRISPR associated protein 9 (CRISPR/Cas9) to engineer *CKX* genes that can facilitate trait improvement. Overall, recent advancements in *CKX* and their role in plant growth, stresses and nutrient accumulation are highlighted, which could be used for chickpea improvement.

## Introduction

Micronutrient malnutrition, often known as “hidden hunger” affects more than half of the world’s population, with pregnant women and children in developing nations bearing the brunt of the burden. According to the World Health Organisation (WHO), more than 2 billion people are suffering from hidden hunger and one of the most important responsible factors for malnutrition is food insecurity which affects almost a billion people worldwide (FAO, 2013). Food security is defined as when all people, at all times, have physical, social and economic access to sufficient, safe and nutritious food that meets their dietary needs and food preferences for active and healthy life (FAO, 2016). This is particularly an issue in developing countries where families cannot afford or get access to sufficient and nutritious food. Each year twenty million infants are born with low body weight. As per the malnutrition report around 150.8 million stunted, 50.5 million wasted, and 38.3 million overweight children under five years of age are found (WHO, 2018; Kumar and Pandey, 2020; Ahmed et al., 2022). The reason for malnutrition is due to the insufficient or poor-quality supply or uptake of nutrition. The consumption of legumes in daily diets, especially in developing countries (Afro-Asian countries), could majorly eradicate protein malnutrition. Legumes could act as a base for the development of many functional foods to promote health benefits in humans (Maphosa and Jideani, 2017; Kumar and Pandey, 2020; Ahmed et al., 2022).

Chickpea (*Cicer arietinum *L.) is the world’s second largest, cool season food legume. It is in high demand owing to its high nutritional value. Chickpea is considered invaluable because it provides food for human consumption and feed to livestock. Owing to these astounding properties of chickpea, its production needs to be enhanced to feed and ensure nutritional health and well-being of world’s population. It is also essential to involve the screening techniques, which is useful to promote the breeding program for increasing the growth of chickpea (Talip et al., 2018). However, various factors hamper the yield of this crop. Climate variability has altered plant physiology in a variety of ways. Multiple stressors on plants are increased as a result of environmental extremes and climate unpredictability (Thornton et al., 2014). Heat stress reduces grain yield and productivity, cold stress causes sterility, and drought stress has a deleterious impact on plant morpho-physiology (Barlow et al., 2015). The generation of reactive oxygen species (ROS) such as hydrogen peroxide (H_2_O_2_), superoxide (.O_2_), and hydroxyl radicals (.OH^−^) are accelerated in plant tissues due to harsh circumstances. In addition to other stress hormones like abscisic acid (ABA), jasmonate, and salicylic acid, cytokinins (CKs) also play a significant role in increasing plant stress tolerance and regulate the action of plants defensive mechanisms. Thus, understanding the roles of cytokinin in plant responses to abiotic stressors is very critical imperative.

The cytokinins have been largely involved in the regulation of plant yield, particularly by influencing the grain related traits i.e., number, size and growth of the root (Yamburenko et al., 2017). Moreover, they are found to be involved in stress regulations (Cortleven et al., 2019) and plant mineral concentration status (Gao et al., 2019). It has also been reported that cytokines negatively affect micronutrient uptake regulations (Gao et al., 2019). Cytokinin dehydrogenase (*CKXs*) is a key enzyme that regulates the cytokinin hormone level in plants (Sakakibara, 2006). It is a small gene family, for instance in Arabidopsis only seven *CKX* genes were reported (Werner et al., 2003). Several studies in Arabidopsis showed that *CKX* genes regulate plant growth and developmental processes including shoot and root development, reproductive meristem activity (Werner et al., 2006; Bartrina et al., 2011), and mineral [phosphorus (P), calcium (Ca), sulfur (S) and microelements like copper (Cu), manganese (Mn), iron (Fe), and zinc (Zn)] accumulations (Werner et al., 2010; Bartrina et al., 2011; Chang et al., 2015). However, related information and functional studies about *CKX* genes in chickpea and their utilization to improve agronomic traits are lacking. Various techniques are being employed to develop a sustainable agriculture system to decrease food insecurity. One such method is Genome editing (GE) for crop improvement that has the potential to create a climate-resilient agriculture system on a global scale (Liu et al., 2013). GE technologies have had a significant impact on plant breeding techniques including new strategies for making rapid and precise changes in crop genomes to protect plants from various challenges and enhance crop outputs (Taranto et al., 2018). In genome editing methods, site-specific endonucleases such as zinc-finger nucleases (ZFNs), transcription activator like effector nucleases (TALENs), and CRISPR-Cas9 are used (Zhu et al., 2017). Unlike the ZENs and TALENs genome editing tools, the CRISPR/Cas9 system is proving to be the most effective GE technology since it is cost-efficient, rapid, accurate, and allows for various site-specific genome editing (Abdelrahman et al., 2018a). In comparison to other genome editing methods like TALENs/ZFNs, CRISPR-based techniques have been intensively investigated in plant genomes. It also offers a lot of potential for assisting crop breeders in developing high-yielding, stress-resistant varieties (Abdelrahman et al., 2018b). Most importantly, CRISPR/Cas9 is coming up as a straight forward, environmentally benign strategy for making genome-edited non-transgenic plants to combat environmental extremes and maintain food security (Haque et al., 2018).

Apart from genome editing tools like CRISPR-Cas9 which are being increasingly used nowadays to develop stress tolerant varieties in different crops, there are some alternative strategies as well. Selection from landraces, hybridization to develop novel variety followed by pedigree selection, mutation breeding, and exploitation of hybrid vigour are examples of traditional breeding procedures that have resulted in considerable improvements in stress tolerance and nutritional quality. Applications of PGPRs are being used to alleviate abiotic stresses and increase productivity in economically important crops including rice, soybean, lettuce, tomato, maize and wheat. Nano-biotechnology has also proven to be a promising tool for sustainable agriculture, seed treatment and germination, plant growth and development, disease diagnostics, and detection of harmful agrochemicals (Nuruzzaman et al., 2016).

In this comprehensive review article, we emphasize the importance of legumes with reference to chickpea, malnutrition and food security, biotic and abiotic stresses, and the role of cytokinin. We also discuss how recently developed genome editing technologies such as CRISPR/Cas9 are being utilized to engineer *CKX* genes to improve agricultural traits and biofortification in chickpea.

## Legumes as Boon to Mankind

Legumes are plants of the Leguminosae/Fabaceae family that bear seeds in pods (Staniak et al., 2014; Kouris-Blazos and Belski, 2016) and as a distinguishing feature fix atmospheric N_2_ in symbiosis with suitable rhizobia. Agriculturally significant legumes fix 40–60 million metric tonnes N_2_, along with an additional 3–5 million tonnes by wild legumes, annually (Smil, 1999). The principal edible legumes are bean, broad bean, chickpea, cowpea, pea, pigeon peaand lentil (National Academy of Science, 1994). However, peas, broad beans, lentils, soybeans, lupins, sprouts, mung bean, green beans, and peanuts are common legumes utilized for human consumption and are known as grain or grain food legumes (Yorgancilar and Bilgicli, 2014). Food legumes are classified into two types: oilseeds and pulses. The oilseeds are high-oil-content legumes such as soybeans and peanuts, whereas the pulses are all dry seeds of cultivated legumes eaten as traditional food. These seeds are recognized globally as a low-cost meat substitute and regarded as the second most important dietary source after cereals (Kouris-Blazos and Belski, 2016). Legumes are high inprotein, essential amino acids, complex carbohydrates, dietary fibre, unsaturated fats, vitamins, and critical minerals, all of which are important in the human diet (Bouchenak and Lamri-Senhadji, 2013; Clark et al., 2014; Rebello et al., 2014). Due to the abundance of useful bioactive chemicals, legumes also have been assigned economic, cultural, physiological, and therapeutic functions in addition to their nutritional excellence.

Legumes are an excellent source of high-quality protein, including 20–45% protein and particularly high in the important amino acid lysine (Philips, 1993). Peas and beans contain 17–20% protein, whilst lupins and soybeans contain 38–45% protein (Mlyneková et al., 2014; Kouris-Blazos and Belski, 2016). Legumes contain twice the protein level of cereals and are richer in protein than most of the other plant diets (Leonard, 2012; FAO, 2016; Kouris-Blazos and Belski, 2016). Leguminous proteins, with the exception of soybean protein, are poor in the important sulphur-containing amino acids namely methionine, cysteine, and cysteine, as well as tryptophan, and are thus regarded as an inadequate source of protein (Kouris-Blazos and Belski, 2016). The primary components of leguminous protein are albumins and globulins, which are further subdivided into vialin and legumin. Vialin is the primary protein group in most of the legumes and is defined by a low quantity of sulphur-containing amino acids, which reflects those legumes have small amounts of sulphur-containing amino acids (FAO, 2016). In terms of protein, legumes and cereals complement each other because cereals are high in sulphur-containing amino acids (poor in legumes) and low in lysine (high in legumes) (Staniak et al., 2014). As a result, when beans are combined with grains, the protein quality improves dramatically (FAO, 2016).

Legumes contain up to 60% carbohydrates by dry weight and are source of complex energy-giving carbohydrates (Leonard, 2012). Leguminous starch digests more slowly than cereal and tuber starch. As a result, beans have a low glycemic index (GI) rating for blood glucose control (Philips, 1993; Khalid and Elharadallou, 2013) making them ideal for diabetic patients and those at greater risk of acquiring diabetes. In general, legumes are beneficial for people who want to live a healthy, disease-free lifestyle (Bouchenak and Lamri-Senhadji, 2013). Legumes are also a rich source of dietary fiber (5–37%), with large levels of both soluble and insoluble fibers (Philips, 1993; Leonard, 2012; Kouris-Blazos and Belski, 2016). Diets high in dietary fiber have been linked to plenty of health advantages. Constipation, obesity, diabetes, heart issues, piles, and various malignancies are among the various diseases and ailments that can be prevented and treated (Maphosa and Jideani, 2016; Tamang et al., 2016).

Except for peanuts (45%) and soybeans (47%), legumes have no cholesterol and are generally low in fat, with 5% calories from fat (Messina, 2016). Legumes have a high concentration of mono- and polyunsaturated fatty acids (PUFA) and almost no saturated fatty acids (Kouris-Blazos and Belski, 2016). Kidney beans and chickpeas have the highest levels of PUFA (71.1%) and MUFA (34%), respectively (Kouris-Blazos and Belski, 2016). Because the human body cannot synthesize these PUFAs, they must be consumed through the diet (FAO, 2016). Legumes are high in B-complex vitamins like foliate, thiamin, and riboflavin, but low in fat-soluble vitamins and vitamin C. (Kouris-Blazos and Belski, 2016). Folate is an important nutrient shown to minimize the likelihood of neural tube abnormalities such as spina bifida in newborns (FAO, 2016; Messina, 2016). Zinc, iron, calcium, selenium, phosphorus, copper, potassium, magnesium, and chromium are also found in legumes (Brigide et al., 2014; Kouris-Blazos and Belski, 2016). These micronutrients serve critical physiological functions in bone health (calcium), enzyme activity and iron metabolism (copper), carbohydrate and lipid metabolism (chromium, zinc), hemoglobin production (iron), antioxidative action, protein synthesis, and plasma membrane stability (zinc) (Mogobe et al., 2015). Legumes are often low in sodium, which is ideal given current developments advocating sodium reduction (Leonard, 2012).

## Chickpea as a Wonder Legume

Chickpea has been identified as the second most important legume and it has numerous economic facilities. According to Chen et al. (2020), India has been identified as the largest chickpea producer with nearly 65% of total global chickpea production. It is being cultivated on about 12 million hectares and its annual production rate is 9 million tonnes. Chickpea contains high protein content, and is also rich in dietary fibres, calcium, zinc, phosphorus and magnesium. Due to a heavy breeding programme, the production rate of chickpea is gradually increasing in the last thirty years. Determination of the effectiveness and importance of yield components have been identified as the main target. According to Coyne et al. (2020), it has been reported that there is a positive relationship between plant height, seed mass, number of pods per plant and number of branches. The grain yield of chickpea is dependent on different quantitative characteristics, such as environmental location and genetic factors.

Chickpea is a true self-pollinated diploid (2 n = 2 x = 16) with an estimated genome size of 738 Mb having 28,269 genes (Varshney et al., 2013). It is the second most economically important pulse crop with the production of 14.24 million metric tons (FAOSTAT, 2019). Chickpea is valuable because it provides both human food and livestock feed and there is a growing demand for chickpea due to its nutritional value. It is an effective source of protein, carbohydrates, minerals and vitamins, dietary fiber, folate, β-carotene, anti-oxidants, micronutrients (phosphorus, calcium, magnesium, iron and zinc) and health-promoting fatty acids (Sharma et al., 2013a; 2013b). It is rich in carbohydrates and proteins, which altogether account for 80% of the total mass of dry seed (Chibbar et al., 2012). Chickpea’s protein content trends vary considerably by17–22% as a % of the total dry seed mass before dehulling and 25.3–28.9% after dehulling (Hulse, 1989; Misra et al., 2016) and is about 2-3 folds more than cereals. The composition of amino acid in chickpea is well balanced having minimal amino acid containing sulphur i.e., methionine, cysteine and a considerable amount of lysine making it an excellent combination with cereals, which are good source of sulfur-containing amino acids. It is rich in carotenoids responsible for the yellow color of the cotyledon. The prominent and widely distributed carotenoid in chickpea is β-carotene and is more efficiently transformed to vitamin A than any other carotenoids. Chickpea has a higher amount of β-carotene on a dry seed weight basis than “golden rice” endosperm or red wheat. It has a higher dietary fiber content (∼18–22 g), particularly in comparison to wheat (∼12.7 g) and a higher fat content, especially in comparison to other pulses or cereals and two polyunsaturated fatty acids (PUFAs) namely, linoleic and oleic acids that constitute approximately ∼50–60% of chickpea fat, therefore works as cholesterol reducer food.

## Abiotic and Biotic Stresses in Chickpea

External factors that negatively affect plant growth, development, or productivity are referred to as stress in plants (Verma et al., 2013). Plants face abiotic and biotic the two main types of stresses and as sessile organisms are continually confronted with a variety of biotic and abiotic stressors. They require continual alterations at the molecular level in order to adapt to changing conditions. Epigenetic regulators provide efficient and effective controls to promote plant survival by increasing their tolerance to stresses (Richards, 2006; Hirayama and Shinozaki, 2010). Different chemical alterations at the molecular level that regulate gene expression are involved in epigenetic control. Today, epigenetics refers primarily to alterations that are related to chemical modifications not to changes in DNA sequence and can be passed on through generations (Feng et al., 2010; Fujimoto et al., 2012). Plants use three types of epigenetic regulatory systems to resist severe conditions caused due to stress conditions: DNA methylation, histone modification, and RNA interference (RNAi). Plants respond to stresses in a variety of ways, including changes in gene expression, cellular metabolism, growth rates, crop yields, and so on. Severe stresses cause crop plants to die by inhibiting flowering, seed development, and inducing senescence (Verma et al., 2013). Abiotic stress is the adverse effect on biological organisms in a particular environment caused by non-living elements. Toxic abiotic stressors, such as hyper drought and salinity, low or high temperatures, depleted or surplus water, high salt levels, heavy metals, and UV radiation are examples of abiotic stress which pose a threat to plant development and growth, resulting in a significant agricultural production penalty throughout the globe. These stresses also adversely affect plant nutrition, for instance when water availability is limited due to drought, total nutrient intake is lowered, and mineral nutrient concentrations in agricultural plants are frequently reduced. Water shortages have the most significant impact on nutrient transport to the root, root growth and extension. The interference of nutrient uptake and unloading mechanisms as well as lower transpiration flow results in reduced nutritional absorption (Marschner 1995; Baligar et al., 2001). Due to lower tissue nutrient concentrations, root development is inhibited when the soil temperature is too low. Nutrient insufficiency may lead to stunted or dying of plant tissue as well as yellowing of leaves. A reduction in crop output or a decline in plant quality growth might be the outcome of nutrients shortage. Traditional and contemporary methods of plant breeding that aim to improve stress tolerance would benefit from a better knowledge of how plants respond to abiotic stresses.

Apart from the abiotic stress, there are many other biotic stresses, such as insect pests, plant-parasitic nematodes and chickpea diseases. Identification of these diseases is accountable for reducing the risk of chickpea cultivation. According to Kumar and Naik (2020), the major disease of chickpeas, such as ascochyta blight (*Ascochyta rabie*), phytophthora root rot (*Phytophthora medicaginis*) and *Botrytis cinerea* is accountable for decreasing the growth of chickpea. There are some major insect pests, such as *Helicoverpa punctigera* and *Helicoverpa armigera,* which are accountable for reducing the nutritive value of chickpea (Abdelrahman et al., 2018a). Moreover, the plant-parasitic nematodes, such as root-lesion nematodes and Crystormin nematodes are accountable for decreasing the 14% growth of chickpea.

The pod borer (*Helicoverpa armigera*) is the most dangerous, followed by the pod fly. Nematodes, which have been efficiently managed by bio-agents, are another key pest impacting chickpea. Wilt and root-knot nematodes are crucial in terms of distribution and chickpea yield damage. Chickpeas are typically grown as rainfed crops since they require less irrigation than competitive crops such as cereal. Post-harvest losses are responsible for 9.5%of total chickpea production. Among post-harvest processes, storage accounts for the greatest amount of loss (7.5%). Processing, threshing, and transportation all result in 1%, 0.50%, and 0.50% losses, respectively. Chickpeas are likewise the most vulnerable to insect damage (5%) among storage losses, compared to wheat (2.5%), rice (2%), and maize (3.5%) (Deshpande and Singh, 2001). Unfortunately, improvements in legumes yield have lagged as those of cereals.

Worldwide, plant productivity is hampered by a lack of water and high salinity. Plants have evolved sophisticated and sensitive defense systems that allow them to signal immediately, respond to, and adapt to a variety of challenges, including drought and excessive salt (Yamaguchi-Shinozaki and Shinozaki, 2006; Tran L. S. P. et al., 2007; Tran L.-S. P. et al., 2007). Plant’s defensive responses to abiotic and biotic stress factors are regulated by various phytohormones.

## Role of Cytokinin in Plant Growth, Stresses and Biofortification in Chickpea

Cytokinins appear to be implicated in stress reactions, according to growing evidence (Tran L.-S. P. et al., 2007; Argueso et al., 2009) and regulate various aspects of root growth, architecture, and function and plays a crucial regulatory role in a variety of developmental and physiological plant processes (Werner and Schmulling, 2009; Hwang et al., 2012). Cytokinins have been identified as a key signal that passes from roots to shoots (Letham, 1994). According to recent research, the abscisic acid (ABA) and cytokinin ratios in xylem sap are critical for stress signaling (Alvarez et al., 2008; Schachtman and Goodger, 2008). Drought, for example, reduces the generation and distribution of cytokinins from roots. The major enzymes involved in cytokinin metabolism in plants such as *Arabidopsis thaliana* are adenosine phosphate-isopentenyltransferases (IPTs) and cytokinin oxidases/dehydrogenases (CKX) (Hirose et al., 2008; Werner and Schmulling, 2009). Cytokinin oxidases/dehydrogenases accelerate irreversible cytokinin breakdown by selectively cleaving unsaturated isoprenoid side chains, culminating in the synthesis of adenine/adenosine and the associated side-chain aldehyde (Sakakibara, 2006; Werner et al., 2006).

Plant cytokinin levels have been altered in genetic experiments assuming usually a negative participant in stress response (Nishiyama et al., 2011). For example, in transgenic tobacco plants, overexpression of the cytokinin degrading enzyme cytokininoxidase/dehydrogenase improved drought and heat stress tolerance (Macková et al., 2013). However, recent research suggests that cytokinins (CK) are N6-substituted adenine derivatives that were first identified as a major regulator in plant developmental processes such as organ formation, apical dominance, leaf senescence (El-Showk et al., 2013) and may play a significant role in drought stress adaption as a positive regulator (Hai et al., 2020). For example, in transgenic cotton (Kuppu et al., 2013), creeping bentgrass (Xu et al., 2016), eggplant (Xiao et al., 2017), and tropical maize (Leta et al., 2016), ectopic expression of the isopentenyltransferase gene (IPT), which encodes a rate-limiting enzyme in cytokinin biosynthesis, increases endogenous cytokinin levels. According to a new rice cytokinin-responsive transcriptome analysis, a substantial number of genes are implicated in both biotic and abiotic stressors (Raines et al., 2016). Temperature, drought, osmotic stress, salinity, nutritional stress, plant diseases, and herbivores are among the environmental conditions where cytokinin is said to be essential for responses (Raines et al., 2016; Cortleven et al., 2019).

Accordingly, multiple functional studies were undertaken to determine the *CKX*-mutant derived tolerance mechanisms. For example, partial root-zone drying resulted in lower cytokinin concentrations in leaves, buds, and shoot tips. This increased apical dominance and aided in overcoming drought stress, particularly when combined with ABA modulation of stomatal apertures. In plants exposed to drought stress, tolerance responses may be induced by manipulating endogenous cytokinin levels, either by deletion of the biosynthesis genes isopentyltransferase or by overexpression of cytokinin oxidase (*CKK*)-encoding degradation genes. Meanwhile, heat stress is known to lower cytokinin levels, and thus exogenous cytokinin application has generally been shown to improve plant heat stress responses, combating the negative effects of heat stress on photosynthesis and chloroplast growth. Additionally, N6-(D2-isopentenyl) adenine (iP) and trans-zeatin (tZ), the biologically active free-base forms of cytokinins, were found to play a key role in tolerance mechanisms, thought to be *via* yielding higher relative abundances and affinities for cytokinin receptors. Thus, *CKX* enzymes play a key role in controlling cytokinin concentrations, which influences plant growth and development. Plants evolve through structural and metabolic adaptations to cope with stress, such as increased root area and leaf curling when subjected to dryness, and increased production of antioxidant chemicals like carotenoids, proline, and ascorbic acid. Plants with bigger root systems have a higher chance of competing for nutrients and surviving in low-nutrient environments (Passioura, 1981; Brown et al., 1989; Saxena et al., 1993; Morita and Nemoto, 1995; Kondo et al., 1999; Steele et al., 2006; Henry, 2013). Root biomass and the availability of soil resources like water and minerals have a big impact on seed output and quality. W31:CaCKX6 expressions in chickpea roots have shown to boost root biomass, shoot biomass and yield (Khandal et al., 2020). The broader root network, obtaining more nutrients from the soil and enhancing the plant’s lifetime, are ascribed to the increased vegetative and reproductive growth of shoots in chickpea lines with W31:CaCKX6.

Chickpea lines expressing W31:CaCKX6 had higher relative water content (RWC) in their leaves, indicating that they were more drought tolerant. Better leaf RWC mixed with lower ABA levels may have contributed to higher carbon assimilation under long-term drought conditions. Chickpea plant introgressed with W31:CaCKX6 in chickpea root produced the seeds having better concentrations of zinc, iron, copper, phosphorus, magnesium, and potassium (Khandal et al., 2020). Thus, increasing the root network through local biofortification of cytokinin and lowering the ABA content employing genome editing in combination with classical breeding may be an effective approach for maintaining the balance for enhanced yield, grain quality and stress tolerance.

### Potentials of Cytokinin Dehydrogenase

A search of the annotated *Medicago truncatula* genome assembly turned up nine CKX-encoding genes (Young et al., 2011). A comparable search of publicly available annotated chickpea genome and transcriptome sequences (Garg et al., 2011; Varshney et al., 2013) revealed the presence of 10 non-redundant genes that encode proteins with sequence similarities to seven Arabidopsis CKX proteins (Schmulling et al., 2003). They were annotated as *C. arietinum* cytokinin oxidases/dehydrogenases (*CaCKX*) and as a result, the chickpea genome has ten *CaCKX* genes. Climate change and population growth have put pressure on the agriculture industry to enhance productivity, resulting in the development of new, improved technologies aimed at improving crop’s ability to remain productive in conditions such as high temperatures and low moisture availability.

### Current Status of *CKX* in Chickpea

The current review highlights the effectiveness of the spatio-temporal regulation of cytokinin, which is significant for nodule development. Investigating the root manipulation for cytokinin is essential for managing the growth of chickpea. Promoter-driven CaWRKY31 in chickpeas, CaCKX6 expression resulted in a larger root system, increased CKX activity in the root, and increased seed yield. With the help of W31:CaCKX6 construct and the chickpea cultivar Pusa 362, T4 transgenic chickpea plants were created (IC296139). Root nodulation and nitrogen fixation were not affected while increasing the CKX activity. When grown in soil rite pots in a controlled growth environment, chickpea transgenic plants showed up to 1.8-fold increase in root length and lateral root numbers in the 10 days post germination (dpg) stage. In soil-grown 30 dpg plants, CKX activity was measured, and only the root showed a 2.1–3.7 fold increase over the untransformed plant, whereas CKX activity in the shoot tissue remained unchanged. The total length of the roots rose by 1.5–1.85 times. The average amount of biomass in the shoots increased by up to 20%. In transgenic chickpea lines, the root-to-shoot biomass ratio was raised by up to 1.7 times. According to two-year growth statistics, average seed number per plant increased by 20%–25%, with no significant variance in 100 seed weight. Statistical significance was often poor due to variance in seed counts between individual plants of a line. CaCKX6 expression in the roots also increased mineral content in seeds like the concentrations of Zn (27 %–62%), Cu (26 %–61%), Fe (22 %–48%) etc. were all greater in transgenic lines’ seeds (Khandal et al., 2020).

Due to the presence of effective nutrients, the global economic demand and importance of chickpea are gradually increasing. It has been detected that chickpea is one of the good sources of multivitamins, such as niacin, riboflavin, thiamin, vitamin A (β carotene) and folate for the fulfilment of nutritional requirements. There are three primary components, such as inadequate supply, food accessibility and inappropriate food, which are accountable for food insecurity. Maintenance of sustainability in the cultivation of chickpea to fulfil nutritional requirements and increase economic values has enhanced food security. However, it has been identified that the production of chickpea is dependent on several challenging situations, different abiotic stresses, such as high and low temperature and drought.

## Biofortification Strategies

Agronomic practices and plant breeding is accountable for providing sufficient nutrients to people. However, one approach towards achieving greater food security is through improving the nutrient value of the food that is consumed. This may be achieved through biofortification approaches *via* breeding for enhanced concentrations of bioavailable nutrients within staple food crops. Several efficacy studies have demonstrated that the biofortification of staple crops can effectively alleviate micronutrient malnutrition or “hidden hunger” among vulnerable populations across the world. Biofortification is one of the innovative techniques, which is used to increase the level of nutrients such as minerals, vitamins and minerals for the enhancement of product’s demand. The nutritional value of legumes including other crops can be increased with the help of various methods such as traditional breeding, molecular technologies, transgenic approaches or genome editing approaches, thereby preventing malnutrition. The former is a well-established, albeit is a labor-intensive and long-term operation.

### Molecular Technology-Assisted Biofortification

The efforts have mainly focused on cereal grain staple species, whereas the application of this approach to grain legume/pulse crops has been largely overlooked. The process of biofortification in agronomic crops includes targeting the cytokinin gene family. The cytokinins are one of the phyto hormones that play essential roles in both physiological and developmental processes and directly impact several agronomic parameters, including growth, development and yield, including root extension and branching during post-embryonic advancement. The root-specific degradation of cytokinin was used to engineer maize genetically (*Zea mays* L.) plants to have a larger root system. Root-specific expression of a cytokinin oxidase (CKK)/dehydrogenase (CKX) gene of *Arabidopsis* caused the formation of up to 46% more root dry weight while shoot growth of the same transgenic lines was similar to the control plants. Meanwhile, the concentrations of K, P, Mo and Zn were significantly increased in the leaves of the transgenic plants. Subsequently, fine-tuning of cytokinin metabolism by root-specific expression of a cytokinin degradation enzyme was undertaken to improve both Zn nutrient level and yield traits.

### Biofortification in Chickpea

**FIGURE 1:**
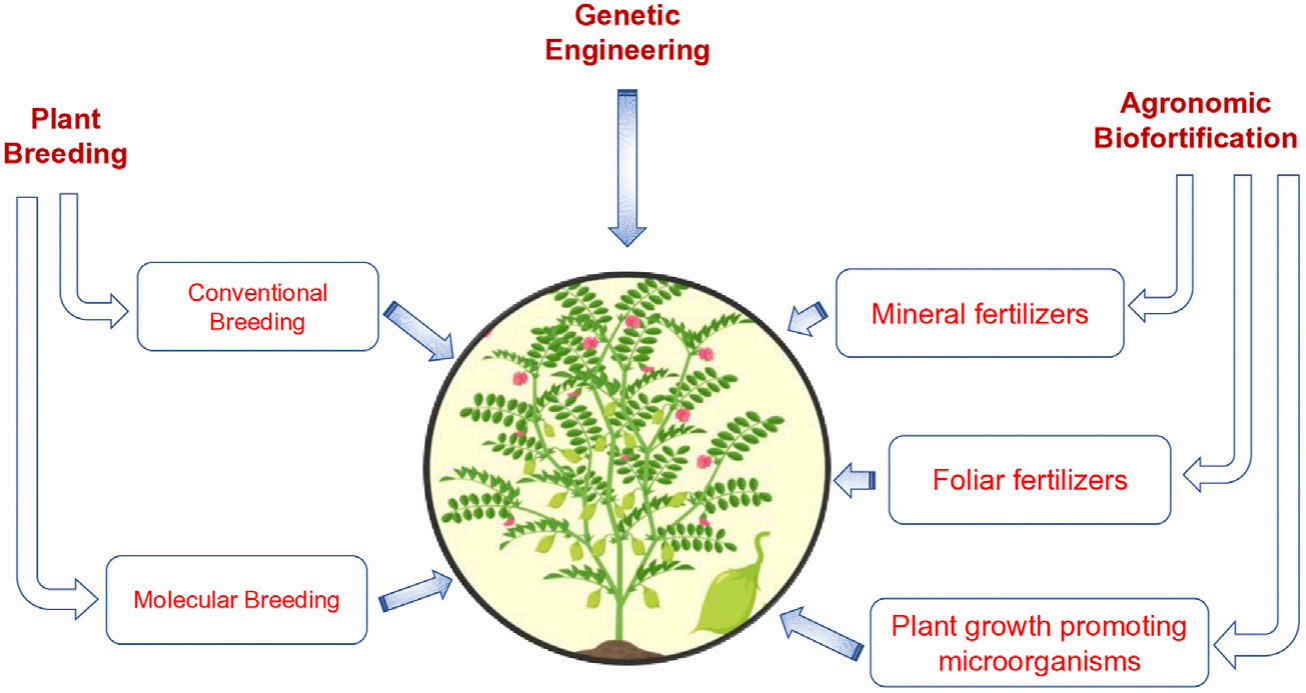
Diagram showing the various approaches to biofortified chickpea.

Chickpea has been identified as one of the effective nutritious crops, for decreasing the negative impact of nutritional deficiencies. According to Yadav et al. (2019), there are nearly 40–60% of low digestible carbohydrates, 4–8% of essential fats, 15–22% of proteins and a sufficient range of vitamins and minerals (Wang et al., 2020). The presence of these nutrients is essential for increasing the nutritional value of chickpeas. Henceforth, the fatty acid composition is accountable for increasing the value of the seed. It has been identified that fat is essential for governing the texture, flavour, shelf life, nutritional composition and aroma. Therefore, the involvement of the biofortification of essential fatty acids is significant for the fulfilment of the nutritive value of crops including chickpea (Abdelrahman et al., 2018b). Some potential area for biofortification in chickpea is given in Figure 1.

Malnutrition has been considered as the cause of global calamity in Asia and Africa. According to Ninan et al. (2019), the current biofortification efforts focus on the enrichment of significant micronutrients and decreasing the anti-nutrient factors. Implementation of the Agronomic approaches, such as fertilizer application is essential for the enrichment of different minerals, such as Zn, Se and Fe. The combined application of Zn, Fe and urea is accountable for increasing the Zn and Fe concentration in the chickpea. It has been detected that the implementation of the transgenic approaches is one of the most efficient for iron biofortification in chickpea. According to Lastochkina, (2019), over expression of the nicotinamide synthesis, such as ferritin (GmFER) and 2 (CaNAS_2_) is essential for increasing the Fe concentration rate in chickpea (Toğay et al., 2019). The biofortification process focused on the macro nutritional traits. Linoleic acid (LA; ω-6) has been identified as the essential fatty acid to facilitate human health. Whereas (α-linolenic acid) ALA is the other most essential fatty acid for managing human health benefits (Talip et al., 2018). It has been identified that there are about 3.8–10.2% of general facts in chickpeas. Enhancement of the nutritional values in chickpea is essential for managing the growth of chickpeas, simultaneously its quality and economic value.

Conversely, developments in molecular technologies based biofortification and the availability of improved species-specific genomic resources have led to the evolution of gene editing methods with targeted precision and validated outcomes within a relatively short time frame. Emerging popular genomic targets for the focus of biofortification efforts in food crop species are members of the cytokinin gene family expression pathway, phytohormones essential for many varied physiological and developmental processes.

## Genetic Engineering of *CKX* Genes

**FIGURE 2:**
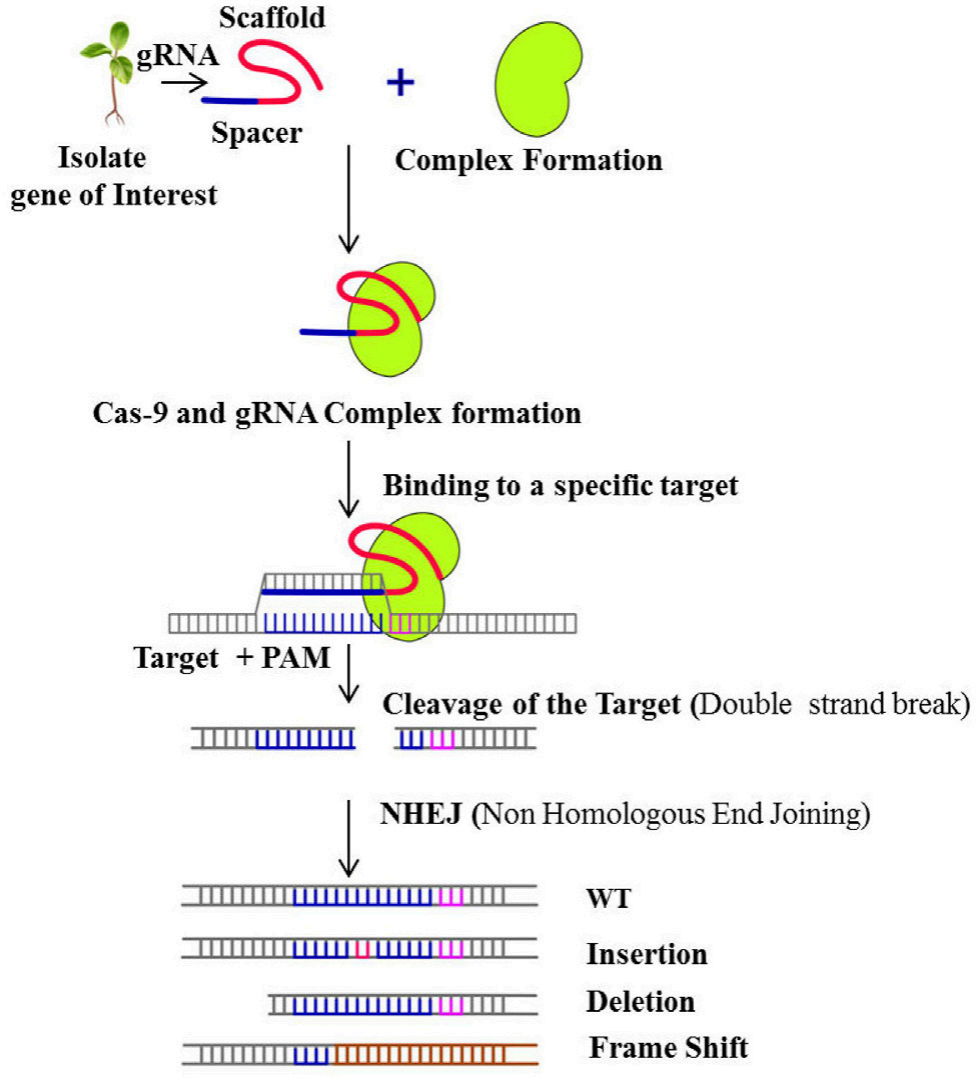
Schematic presentation of mechanism of CRISPR/Cas-9 gene editing technology.

Genomic editing or genetic engineering is an important aspect of today’s world, in which the DNA is inserted, modified or deleted. First genome editing technologies were developed in the 1900s (Kiran and Chimmad, 2018). These technologies act like scissors and cut the DNA at specific sites. The most efficient tool for genome editing is the CRISPR/cas9 system. This system is mainly used in the production of genetically modified organisms (GMOs) and genomic engineering. CRISPR/cas9 has extended the scope of agricultural research allowing for new potentials to generate novel plant types with undesirable features removed or significant characters added such as acrylamide-free potatoes (Halterman et al., 2015); non-browning apples, mushrooms and potatoes; low phytic acid maize (Liang et al., 2014); blast disease resistant rice (Wang et al., 2016) and powdery mildew resistant wheat (Wang et al., 2014). CRISPR technology is continually improving, allowing for more genetic manipulations such as creating knockouts, precise changes, multiplex genome engineering, and target gene activation and repression. With more precision and simplicity, CRISPR targets endogenous genes that are unable to target specifically using RNAi technology the mechanism of which can beseen in Figure 2. CRISPR/Cas9 uses a 100 nucleotide (nt) guide RNA (gRNA) sequence to target specific genomic loci. Using Watson and Crick base pairing through 17–20 nt at the gRNA 5′-end, sgRNA binds to the protospacer adjacent motif (PAM) on targeted DNA and guides Cas9 for selective cleavage. Cas9 accelerates DNA repair by causing DSBs (Double Stranded Breaks) in the target DNA. To induce genomic changes, gene knockouts, and gene insertions, the repair mechanism uses error-prone non-homologous end joining (NHEJ) or homologous recombination (HR). NHEJ makes random insertions or deletions in the coding area, resulting in frame shift mutations and gene knockouts. Thus, loss-of-function, gain-of-function, and gene expression analysis are possible with CRISPR technology enabling it as one of the effective plant breeding tool, which focuses on different gene action by acquiring knowledge on gene family members (GFMs) and is desperately needed.

**FIGURE 3:**
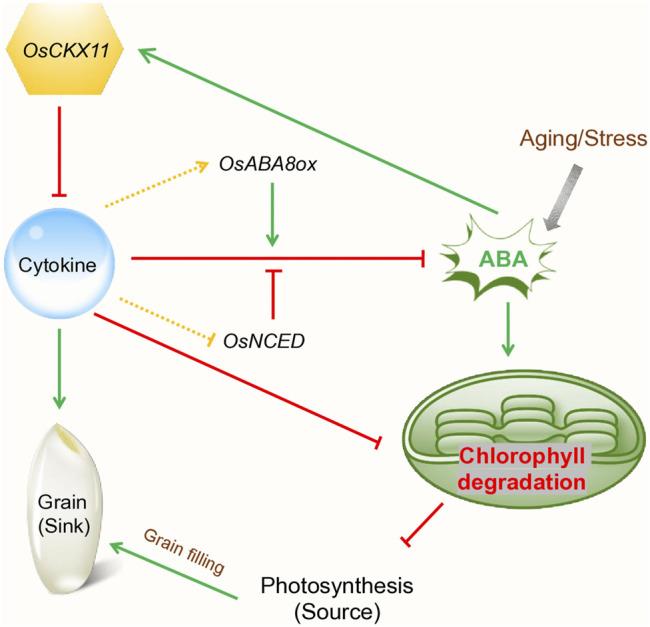
Schematic presentation showing the role of *OsCKX11* in cytokinin-mediated leaf senescence and grain number in rice (Modified from Zhang et al., 2021)

According to Kumar et al. (2017),CRISPR/Cas9is known as one of the effective gene-editing tools, which is accountable for manipulating cytokinin dehydrogenase (Kumar et al., 2017; Jha et al., 2018). The GFMs expression is essential for cytokine biosynthesis and destruction for managing the gene factors. According to Ninan et al. (2019), cytokines have been identified as the enhancement of sink activities in chickpea leaves. The primary steps in cytokine biosynthesis can be controlled by isopentenyltransferase (IPT). Henceforth, the cytokinin dehydrogenase or oxidase is accountable to control the process of cytokinin degradation. Involvement of the DNA sequencing technology is essential for gathering delta knowledge in the gene concept. Cytokinin is identified as one of the effective plant hormones, which is accountable for regulating plant development. The *PsCKX7* (*Pisum sativum *cytokinin dehydrogenase) gene when down-regulated, cytokinin levels increased in roots, shoots and leaves also involves delaying of senescence. It is noteworthy that *PsCKX5* and *PsCKX7* express in the sink and mature leaves respectively (Ninan et al., 2019). In rice, Zhang et al. (2021) performed CRISPR/Cas9 editing to target serval *CKX* genes. They found that *OsCKX11* (*Oryza sativa* cytokinin dehydrogenase) have simultaneously regulates cytokinin-mediated leaf senescence and grain number (Figure 3).

The cytokine dehydrogenase (*CKXs*) is known as an essential protein for an irreversible breakdown of cytokinin’s. It is significant for the molecular evolution for the determination of the homologous protein. Uses of these gene-editing tools are essential for detecting the presence of *CKX* in prokaryotic and eukaryotic. Apart from this, it has been identified that *CKX* plays a significant role in the improvement of plant life. Controlling the plant development process is beneficial for managing the abiotic and biotic stress for influencing the nutritive value of chickpea (Abdelrahman et al., 2018a). Cytokinin dehydrogenase is an essential plant hormone for promoting cell division. Promotion of primary cell growth and differentiation is essential for increasing the growth of this hormone. The involvement of the gene-editing tools helped in gene formation for improving the growth of its products. Apart from cytokinin, ethylene exhibition is equally important for managing the growth of plants. Involvement of the photosynthetic machinery process is essential for stimulating the growth of chickpea (Jyothi et al., 2018). Cytokinin is accountable for increasing the grain size and grain numbers for yielding the components of this plant.

In recent years, the Clustered Regularly Interspaced Short Palindromic Repeats Cas9 (CRISPR/Cas9) genome editing method has revolutionized targeted gene editing in plants (Woo et al., 2015; Baek et al., 2016; Malnoy et al., 2016; Liang et al., 2017; Kim et al., 2018; Lin et al., 2018; Murovec et al., 2018; Osakabe et al., 2018; Johansen et al., 2019; Petersen et al., 2019). CRISPR/Cas9 genome editing has a wide range of applications in agricultural improvement, including the development of designer genetically modified non-GM crops. The application of this strategy to plant breeding for the production of new crop varieties with greater tolerance to environmental challenges is a major focus of agricultural scientists (Khatodia et al., 2016; Noman et al., 2016). CRISPR/Cas9 gene-editing tools have been utilized for gene activation, repression, knockout, knockdown, repression, and for altering epigenetic modifications in several plants crops such as Arabidopsis (Feng et al., 2014), apple (Osakabe et al., 2018), citrus, carrot (Klimek-Chodacka et al., 2018), grape (Nakajima et al., 2017), tomato (Wang et al., 2019), rice (Zhang et al., 2014), sorghum (Liu et al., 2019), maize and soybean (Chilcoat et al., 2017), and wheat (Zhang et al., 2016). CRISPR/Cas9 genome editing was utilized to discover abiotic stress response in Arabidopsis plants; the findings revealed that *OST2* (proton pump), a mutant allele produced through editing, changed stomatal closure under environmental stress (Osakabe et al., 2018). Another recent maize work employed the CRISPR/Cas9 method to create unique allelic variants that could be exploited to engineer drought-tolerant crops. This system genetically modified *ARGOS8*, whose over expression can result in lower ethylene sensitivity. Field investigations demonstrated that *ARGOS8* variants had higher grain yield under drought stress; further, no yield loss was documented under well-watered conditions (Shi et al., 2017).

## Conclusion and Future Prospects

This present review highlights the role of *CKX* genes in chickpea growth and development traits, biotic and abiotic stress regulation, and biofortification. Chickpea is the most economically important product all over the world. There are various types of stress like heat, cold, drought and so on those are faced by the crop plant. Due to global warming, a temperature rise is a frequent event in today’s world that causes the drought condition. Heat stress causes severe damage to the leaves and also ruptures the membrane. All these factors adversely affect the agronomic traits of chickpea. CKs play many crucial roles in plants when they experience any kind of stress. Phytohormones in the cytokinin family control root length and branching in the post-embryonic stages. Cytokinin oxidases or dehydrogenases (CKXs) are enzymes that degrade cytokinin in order to study its biological functions and engineer root development. A chickpea root-specific promoter of CaWRKY31 may be used to explore how cytokinin depletion affects root development and drought tolerance in *Arabidopsis thaliana* and chickpea with definite and indeterminate growth patterns, respectively. In *Arabidopsis* and chickpea, root specific expressions of CaCKX6 increased lateral root number and plant biomass without affecting shoot vegetative and reproductive development. Root CKX activity was elevated in transgenic chickpea lines. The root-to-shoot biomass ratio was greater in soil-grown advanced chickpea transgenic lines, and the plants had improved long-term drought resistance. Nutrient fixation in the roots and leaves of these chickpea varieties was unaffected. In certain transgenic lines, the seed output was up to 25% greater with enhanced concentrations of zinc, iron, potassium, and copper without corresponding decrease in protein content. Apart from this other phytohormones also play an important role in alleviating stress condition in chickpea. ABA plays an important role to reduce oxidative damage in chickpeas. It interacts with the various types of antioxidants to reduce stress and reduces the ROS production in the plant body which harms the plants. It has also introduced some heat shock protein to provide tolerance against the heat. Salicylic acid also plays an important role against abiotic and biotic stress as well as against pathogens and herbivores. However, in chickpea, functional characterization studies of *CKX* genes have just started. Gene editing tools such as TALENs or CRISPR/Cas9 can play crucial role in this context. Still, less functional studies exist in the case of stress regulation and biofortification. This is a potential area for research to unravel the CK signaling networks and their cross talk elucidating its biochemical pathways which will draw a detailed picture and pave the road towards developing tolerant crops, and in the long-term, more sustainable agriculture. Similarly, many more such genes are hidden in the plant genome, which are required to be explored and investigated to harness and develop cultivars with a higher yield, better abiotic stress resistance and biofortification.

## Data Availability

The original contributions presented in the study are included in the article/Supplementary Material, further inquiries can be directed to the corresponding author.
